# The Association of Vision, Hearing, and Dual Sensory Impairments With Walking Speed and Incident Slow Walking

**DOI:** 10.1093/geroni/igaa057.698

**Published:** 2020-12-16

**Authors:** Ahmed Shakarchi, Varshini Varadaraj, Lama Assi, Nicholas Reed, Bonnielin Swenor

**Affiliations:** 1 Johns Hopkins Wilmer Eye Institute, Baltimore, Maryland, United States; 2 Johns Hopkins University, Baltimore, Maryland, United States

## Abstract

Vision (VI), hearing (HI) and dual sensory (DSI, concurrent VI and HI) impairments are increasing in prevalence as populations age. Walking speed is an established health indicator associated with adverse outcomes, including mortality. Using the population-based Health and Retirement Study, we analyzed the longitudinal relationship between sensory impairment and walking speed. In multivariable mixed-effects linear models, we found differences in baseline walking speed (m/s) by sensory impairment: Beta=-0.05 (95%CI=-0.07, -0.04), Beta=-0.02 (95%CI=--0.03, -0.003), and Beta=-0.07 (95%CI=--0.08, -0.05) for VI, HI and DSI, respectively, as compared to those without sensory impairment. However, similar annual declines (0.014 m/s) in walking speeds occurred in all groups. In time-to-event analyses, events were defined as “slow walking” (speed <0.60m/s) and “very slow walking” (<0.40m/s). Incident “slow walking” was 43% (95%CI=25%, 65%), 29% (95%CI=13%, 48%) and 35% (95%CI=13%, 61%) greater in VI, HI and DSI, respectively, than the no sensory impairment group, while incident “very slow walking” was 21% (95%CI=-4%, 54%), 30% (95%CI=3%, 63%) and 89% (95%CI=47%, 143%) greater; the increase was significantly greater in DSI than VI and HI. These results suggest that older adults with vision and hearing impairments walk slower and are at increased risk of slow walking than older adults without these sensory impairments. Additionally, older adults with DSI are at greatest risk of very slow walking.

